# Effects of Negative Emotions and Life Events on Women's Missed Miscarriage

**Published:** 2018-02

**Authors:** Huilin XING, Yaping LUO, Shouying WANG

**Affiliations:** 1. School of Basic Medical Sciences, Xinxiang Medical University, Xinxiang, Henan 453000, P.R. China; 2. Dept. of Gynaecology and Obstetrics, Xiamen Maternal and Child Health Care Hospital, Xiamen, Fujian 361003, P.R. China; 3. Management Institute, Xinxiang Medical University, Xinxiang, Henan 453000, P.R. China

**Keywords:** Negative emotions, Life events, Missed miscarriage, Anxiety, Depression

## Abstract

**Background:**

To investigate the effects of negative emotions and life events on women's missed miscarriage.

**Methods:**

Overall, 214 women diagnosed with a missed miscarriage by prenatal examination from 2016–2017 in Xiamen Maternal and Child Health Care Hospital, Xiamen, China were selected as the observation group compared to 214 women as control group. The general data of the patients were investigated by self-programmed questionnaires. Zung Self-Rating Anxiety Scale, Center Epidemiological Studies Depression Scale; Life Events Scale for Pregnant Women were used conduct the study. General data, anxiety, depression and life events were compared between the two groups of patients, and statistically different factors were included in the multivariate Logistic regression analysis.

**Results:**

There were statistically significant differences in the educational level, pre-pregnancy health status, planned pregnancy, pre-pregnancy or gestational gynecological inflammation and the initiative to obtain knowledge of prenatal and postnatal care between the two groups of pregnant women (*P*<0.01); there were also statistically significant differences in score of life events, score of anxiety and score of depression between them (*P*<0.01). The high educational level, good health status before pregnancy and the initiative to obtain the knowledge of prenatal and postnatal care were taken as the independent protective factors for the missed miscarriage in pregnant women, while life events, anxiety and depression were independent risk factors for it.

**Conclusion:**

Negative emotions and life events increase the risk of women's missed miscarriage, and the high educational level, good health status before pregnancy and the initiative to obtain the knowledge of prenatal and postnatal care reduce the risk of women's missed miscarriage.

## Introduction

The missed miscarriage refers to the phenomenon that the embryo stops developing or dies in the uterus in the early phase (1). In recent years, with the changes in lifestyle and the accelerated pace of life, the clinical emergence of women's missed miscarriage has been gradually increasing, posing great physical and psychological harms to pregnant women and their families (2).

The cause and mechanism of missed miscarriage are very complex, closely related to immune function, infection, inheritance, endocrine, uterine disease, lifestyle, environment and other factors (3). However, the causes of the missed miscarriage in 40%∼50% pregnant women are still unknown (4). Pregnant women's stress, anxiety, depression and other negative emotions can affect the growth and development of the fetus (5).

The study aimed to investigate the effects of negative emotions and life events on women's missed miscarriage.

## Methods

Overall, 214 women diagnosed with a missed miscarriage by prenatal examination from July 2016 to June 2017 in Xiamen Maternal and Child Health Care Hospital, China were selected as the observation group, and 214 women receiving prenatal examination and delivering full-term normal neonatal newborns were selected as the control group. These patients were allocated at the ratio of 1:1 according to age (± 1year old). In the observation group, women with a missed miscarriage due to maternal and fetal rejection, uterine abnormalities, chromosomal abnormalities and endocrine abnormalities were excluded; in the control group, women with the past history of a missed miscarriage were excluded.

All pregnant women in this study signed the informed consent, and this experiment was approved by the Ethics Committee of Xiamen Maternal and Child Health Care Hospital.

The investigation was conducted by gynecologists receiving unified trainings. The general data of patients, including age, body mass index, occupation, educational level, pre-pregnancy health status, planned pregnancy, pre-pregnancy or pregnancy gynecological inflammation, pre-pregnancy physical exercise and supplement with folic acid, were investigated using self-programmed questionnaires. Zung Self-Rating Anxiety Scale (SAS) (6) was used to assess the degree of anxiety in pregnant women. The scale consists of 20 items with four-grade scores, obtaining raw scores of 20 items, which were then transformed into standard scores by multiplication by 1.25, and some items must be reversely scored. The score over 50 points indicates the existence of anxiety, and the higher the score of pregnant women, the more serious the anxiety is. The Center Epidemiological Studies Depression Scale (CES-D) (7) was used to assess the degree of depression in pregnant women. The scale was compiled by Sirodff of the National Institute of Mental Health in 1977. Compared with other self-rating depression scales, CES-D is more focused on the individual's emotional experience. CES-D includes a total of 20 items, and is divided into 4-grade scores according to the frequency. The score more than 20 points represents the obvious existence of depression. Life Events Scale for Pregnant Women (LESPW) (7) was used to assess the patient's life events during pregnancy. The scale includes objective events (OE) and subjective events (SE). OE events are divided into OE1, OE2 and OE3 according to the intensity of emergency.

### Statistical analysis

The data were processed by SPSS 17.0 (Chicago, IL, USA). Measurement data were expressed as x̅ ± s and detected using *t-*test. The χ^2^ test was used for count data. Logistic regression analysis was used for the multivariate analysis. *P*<0.05 represented that the difference was statistically significant.

## Results

There were statistically significant differences in the educational level, pre-pregnancy health status, planned pregnancy, pre-pregnancy or pregnancy gynecological inflammation, the initiative to obtain the knowledge of prenatal and postnatal care (*P*<0.01) (Table 1).

**Table 1:** Comparisons of general data between the two groups of patients

***Item***	***Observation group (n=214)***	***Control group (n=214)***	***Statistical magnitude***	**P**
Age (yr)	28.6±4.7	27.9±4.2	0.579	0.483
Body mass index (kg/m^2^)	23.1±2.9	23.3±3.1	0.206	0.827
Occupation			0.381	0.537
Peasant	23	29		
Workman	40	38		
Commercial personnel	25	31		
Technician	56	52		
Public institution personnel	43	30		
Self-employer	27	34		
Educational level			40.064	0.000
Junior high school and below	112	45		
Senior high school	43	63		
Junior college and above	59	106		
Pre-pregnancy health status			27.925	0.000
Good	165	203		
Normal or poor	49	11		
Planned pregnancy			13.013	0.000
Yes	177	201		
No	37	13		
Pre-pregnancy or pregnancy gynecological inflammation			18.345	0.000
Yes	52	19		
No	162	195		
Pre-pregnancy physical exercise			0.601	0.438
Yes	112	120		
No	102	94		
Folic acid supplement			1.037	0.308
Yes	192	198		
No	22	16		
The initiative to obtain the knowledge of prenatal and postnatal care			12.317	0.000
Yes	157	186		
No	57	28		

There were significant differences in the score of life events, anxiety score and depression score between the two groups of patients (*P*<0.01) (Table 2).

**Table 2:** Comparisons of negative emotions and life events between the two groups of patients

***Item***	***Observation group (n=214)***	***Control group (n=214)***	**t**	**P**
Life events				
OE1	33.1±10.8	24.1±9.7	5.034	0.000
OE2	74.3±21.4	25.8±8.2	22.476	0.000
OE3	136.7±49.5	72.4±20.6	15.148	0.000
SE	17.1±5.4	28.0±9.3	7.307	0.000
LESPW total score	261.2±85.5	150.3±41.6	12.980	0.000
SAS score	45.3±8.8	36.1±8.0	4.425	0.000
CES-D score	19.7±6.5	9.5±5.4	7.438	0.000

The multivariate Logistic regression analysis results showed that high educational level, good pre-pregnancy health status and the initiative to obtain the knowledge of prenatal and postnatal care were independent protective factors for the occurrence of missed miscarriage in pregnant women, while life events, anxiety and depression were independent risk factors for it (Table 3).

**Table 3:** The multivariate Logistic regression analysis

***Influencing factor***	***Coefficient***	***Standard error***	***Wald-c2***	**P**	***Odds ratio (OR)***	***95% confidence interval (95% CI)***
High educational level	−0.794	0.065	149.642	0.000	0.452	0.398∼0.513
Good pre-pregnancy health status	−1.103	−0.497	4.915	0.027	0.332	0.880∼0.125
The initiative to obtain the knowledge of prenatal and postnatal care	−1.201	0.599	4.014	0.045	0.301	0.093∼0.974
Life events	0.880	0.377	5.467	0.019	2.412	1.153∼5.046
Anxiety	0.520	0.219	5.614	0.018	1.682	1.094∼2.586
Depression	0.794	0.314	6.384	0.012	2.213	1.195∼4.098

## Discussion

The missed miscarriage refers to the pathological pregnancy phenomenon that the embryo dying of defections in fertilized ova or adverse factors existing in the matrix stays in the uterine cavity and is not naturally excreted (8). Missed miscarriages accounted for 10% to 18% of early pregnancy (9). Causes for the missed miscarriage are very complex; in addition to genetic and environmental factors, anxiety, depression and other negative emotions may also affect the growth and development of the fetus by influencing maternal and fetal endocrines (10). The incidence rates of preterm delivery, abortion, fetal mental retardation and fetal malformations in pregnant women with severe stress and anxiety are significantly increased (11). Relatively high anxiety and depression levels during pregnancy are independent risk factors for the occurrence of adverse pregnancy outcomes (12).

Mental state is the leading risk factor for the missed miscarriage (13). The results of this study showed that the score of life events, anxiety score and depression score of pregnant women in the observation group were significantly higher than those in the control group. The results of multivariate Logistic regression analysis showed that life events, anxiety and depression were independent risk factors for the missed miscarriage in pregnant women. Most of pregnant women with a missed miscarriage have experienced abortions for more than one time, which leads to greater psychological stress, proneness to emotional stress and psychological long-term stress (14). This is more likely to lead to the occurrence of missed miscarriage, thus leading to repeated abortion. Repeated abortion will cause great psychological trauma to pregnant women, and this psychological trauma will affect the psychological state of the subsequent pregnancy, thus forming a vicious circle. Life events will also be taken as a stress source affecting the psychological state of pregnant women, thus increasing the risk of the occurrence of the missed miscarriage.

The results of this study showed that there were statistically significant differences in the educational level, pre-pregnancy health status, planned pregnancy, pre-pregnancy or pregnancy gynecological inflammation, the initiative to obtain the knowledge of prenatal and postnatal care between the two groups of pregnant women. The multivariate Logistic regression analysis results revealed that the high educational level, good pre-pregnancy health status and the initiative to obtain the knowledge of prenatal and postnatal care were independent protective factors for the occurrence of the missed miscarriage in pregnant women. In general, the educational level of the women in the observation group was lower than that in the control group; women with low educational level were deeply influenced by the traditional concept of child-bearing so that they regarded the continuity of a clan as the main value of their own existence (15), and their psychological burdens were large, easily causing anxiety and depression. In addition, the income of women with a low educational level was relatively low. Women with low disposable incomes have a higher degree of depression than those with high disposable incomes (16). Good health is the basis for a good pregnancy outcome, so the incidence rate of missed miscarriage in pregnant women with good pre-pregnancy health status is relatively low. Pregnant women taking the initiative to obtain the knowledge of prenatal and postnatal care can enhance the precaution consciousness of a variety of dangerous conditions, thus reducing the occurrence of missed miscarriage.

Effective psychological interventions can significantly reduce anxiety, depression and other negative emotions during pregnancy (17). In addition to routine treatments, the psychological counseling needs to be conducted for pregnant women with the history of a missed miscarriage, and psychological supports need to be provided to adjust the psychological state, which are of great values for a good outcome of pregnancy. Besides, for pregnant women preparing for pregnancy, maintaining a good physical condition before pregnancy, taking the initiative to obtain the knowledge of prenatal and postnatal care, keeping a relaxed mood and reducing mental stresses are conducive to getting a good pregnancy outcome.

## Conclusion

Negative emotions and life events will increase the risk of women's missed miscarriage, but the high educational level, good pre-pregnancy health status and the initiative to obtain the knowledge of prenatal and postnatal care will reduce it.

## Ethical considerations

Ethical issues (Including plagiarism, informed consent, misconduct, data fabrication and/or falsification, double publication and/or submission, redundancy, etc.) have been completely observed by the authors.
